# Metabolic, hormonal and performance effects of isomaltulose ingestion before prolonged aerobic exercise: a double-blind, randomised, cross-over trial

**DOI:** 10.1186/s12970-021-00439-z

**Published:** 2021-05-17

**Authors:** Hannah L. Notbohm, Joshua F. Feuerbacher, Finn Papendorf, Nils Friese, Mats W. Jacobs, Hans-Georg Predel, Jonas Zacher, Wilhelm Bloch, Moritz Schumann

**Affiliations:** 1 Department of Molecular and Cellular Sports Medicine, German Sport University Cologne, Am Sportpark Müngersdorf 6, 50933 Cologne, Germany; 2 Department of Preventative and Rehabilitative Sports and Performance Medicine, German Sport University Cologne, Cologne, Germany

**Keywords:** Glucose, GIP, Insulin, Fat oxidation, Glycaemic index, Endurance exercise, Carbohydrate oxidation, Running

## Abstract

**Background:**

Isomaltulose has been discussed as a low glycaemic carbohydrate but evidence concerning performance benefits and physiological responses has produced varying results. Therefore, we primarily aimed to investigate the effects of isomaltulose ingestion compared to glucose and maltodextrin on fat and carbohydrate oxidation rates, blood glucose levels and serum hormone concentrations of insulin and glucose-dependent insulinotropic polypeptide (GIP). As secondary aims, we assessed running performance and gastrointestinal discomfort.

**Methods:**

Twenty-one male recreational endurance runners performed a 70-min constant load trial at 70% maximal running speed (V_max_), followed by a time to exhaustion (TTE) test at 85% V_max_ after ingesting either 50 g isomaltulose, maltodextrin or glucose. Fat and carbohydrate oxidation rates were calculated from spiroergometric data. Venous blood samples for measurement of GIP and insulin were drawn before, after the constant load trial and after the TTE. Capillary blood samples for glucose concentrations and subjective feeling of gastrointestinal discomfort were collected every 10 min during the constant load trial.

**Results:**

No between-condition differences were observed in the area under the curve analysis of fat (*p* = 0.576) and carbohydrate oxidation rates (*p* = 0.887). Isomaltulose ingestion led to lower baseline postprandial concentrations of blood glucose compared to maltodextrin (percent change [95% confidence interval], − 16.7% [− 21.8,-11.6], *p* < 0.001) and glucose (− 11.5% [− 17.3,-5.7], *p* = 0.001). Similarly, insulin and GIP concentrations were also lower following isomaltulose ingestion compared to maltodextrin (− 40.3% [− 50.5,-30.0], *p* = 0.001 and − 69.1% [− 74.3,-63.8], *p* < 0.001, respectively) and glucose (− 32.6% [− 43.9,-21.2], *p* = 0.012 and − 55.8% [− 70.7,-40.9], *p* < 0.001, respectively). Furthermore, glucose fluctuation was lower after isomaltulose ingestion compared to maltodextrin (− 26.0% [− 34.2,-17.8], *p* < 0.001) and glucose (− 17.4% [− 29.1,-5.6], *p* < 0.001). However, during and after exercise, no between-condition differences for glucose (*p* = 0.872), insulin (*p* = 0.503) and GIP (*p* = 0.244) were observed. No between-condition differences were found for TTE (*p* = 0.876) or gastrointestinal discomfort (*p* = 0.119).

**Conclusion:**

Isomaltulose ingestion led to lower baseline postprandial concentrations of glucose, insulin and GIP compared to maltodextrin and glucose. Consequently, blood glucose fluctuations were lower during treadmill running after isomaltulose ingestion, while no between-condition differences were observed for CHO and fat oxidation rates, treadmill running performance and gastrointestinal discomfort. Further research is required to provide specific guidelines on supplementing isomaltulose in performance and health settings.

## Introduction

For prolonged endurance exercise, carbohydrates represent one of the main energy sources [1, 2]. Carbohydrates differ in respect to their glycaemic index (GI), i.e. they are classified based on their postprandial glucose responses [3]. Food sources with a lower GI show lower postprandial glucose concentrations, as well as a decreased insulin response [4]. Furthermore, insulin is known to be a major suppressor of fat oxidation [5], therefore increased fat oxidation rates after low-glycaemic meals before exercise have been observed [6]. Consequently, low GI carbohydrate sources have been suggested to be beneficial for endurance performance due to the preservation of muscle glycogen, sustained carbohydrate availability and maintenance of euglycaemia [4, 6–8], which have been shown to prevent central fatigue [9].

One of the recently investigated low GI carbohydrate sources is isomaltulose, which is a dissacharide composed of α-1,6-linked glucose and fructose. The rate of hydrolysis and absorption compared to sucrose is considerably reduced (up to 85%) [10] due to the α-1,6-glycosidic bond, however, a full absorption is still achievable [11]. Results regarding altered substrate utilisation during exercise after isomaltulose ingestion remain ambiguous. Some studies reported increased fat oxidation and lower carbohydrate oxidation at least at some point during cycling trials (150 min at 50% Wmax [12] or 90 min at 60% VO2peak [13]) while others showed no difference in a short incremental running trial in persons with Type I Diabetes (T1DM) [14].

Effects of isomaltulose ingestion on resting blood glucose and hormone concentrations have been well studied, however in exercise settings, especially in healthy runners, knowledge on these effects is more limited. Oral administration of isomaltulose has previously been shown to result in lower postprandial blood glucose and insulin concentrations in healthy and type 2 diabetes mellitus (T2DM) patients [11, 15–17]. During exercise, insulin concentrations were shown to be lower following isomaltulose ingestion throughout a 150 min cycling trial [18] or not differ during 60 min of cycling [19] compared to sucrose or maltodextrin respectively. The associations of blood glucose, insulin and GIP secretion after isomaltulose ingestion have been studied in resting conditions [16, 20], however to the best of our knowledge these kinetics have not yet been investigated in exercise settings. A reduced secretion of insulin and GIP during exercise would be beneficial to maintain constant blood glucose concentrations and prevent hyperinsulinaemia. Consequently, these possible effects may also explain differences observed in altered substrate utilisation and improved performance in prolonged endurance exercise when comparing isomaltulose and sucrose energy substrates.

However, also only a few exercise studies have focused on endurance performance and substrate utilisation after isomaltulose ingestion in both healthy and T1DM individuals. The results have been inconclusive, possibly because the glycaemic index or load has previously been shown not to affect endurance performance or metabolic responses, when conditions were matched for carbohydrate and energy [21]. Nevertheless, when matched for carbohydrate intake, one study has found improved time trial performance after cycling for 90 min at 70% VO_2_max following isomaltulose ingestion [13], while others have found no difference in cycling and soccer specific exercise [14, 19, 22] or even impaired cycling performance [12]. In addition, due to the slow absorption rate in the small intestine, isomaltulose has been suggested to cause gastrointestinal discomfort when ingested during 2 h cycling at 60% Wmax [12], however other trials have produced contrasting results, when ≤50 g were ingested before cycling [19] or soccer specific exercise [22]. These inconsistent findings may be partly explained by differences in the timing and the amount of oral administration, as well as the type and intensity of exercise and the training status of the participants.

Collectively, this data shows that studies assessing the impact of isomaltulose ingestion on metabolic, hormonal and performance effects during prolonged running in healthy trained individuals are lacking. However, specifically submaximal running exercise can induce a higher oxygen uptake and likely a higher energy expenditure compared to cycling [23–25] and therefore may differ in substrate utilisation. Thus, isomaltulose ingestion could be especially beneficial for endurance runners. Furthermore, gastrointestinal tolerance may be different in running compared to cycling, since runners are generally more prone to experience symptoms of gastrointestinal discomfort compared to cyclists [26].

Accordingly, the objective of this study was to assess the effects of isomaltulose on prolonged running exercise. Thus, our primary aim was to investigate the effects of isomaltulose ingestion compared to glucose and maltodextrin on metabolic and hormonal responses, as assessed by means of fat and carbohydrate oxidation rates, blood glucose levels and serum hormone concentrations of insulin and GIP. As secondary aims, we assessed how these possible metabolic and hormonal changes may impact treadmill running performance and gastrointestinal discomfort.

## Methods

### Participants

Twenty-one male recreational endurance runners (age: 26.2 ± 5.8 yrs., height: 179.2 ± 5.0 cm, body mass: 70.3 ± 5.9 kg, VO_2_peak: 59.5 ± 6.0 ml·min^− 1^·kg^− 1^) participated in this study. Prior to all testing, the medical history of all participants was assessed through a standardised questionnaire and a resting ECG was reviewed by a cardiologist to ensure all participants were healthy and physically fit to complete the experimental trial. Participants were informed about possible risks of study procedures and provided their written informed consent prior to inclusion into the study. The study was conducted in accordance with the declaration of Helsinki and approved by the university’s ethical committee (09/2020).

### Experimental design

**Fig. 1:**
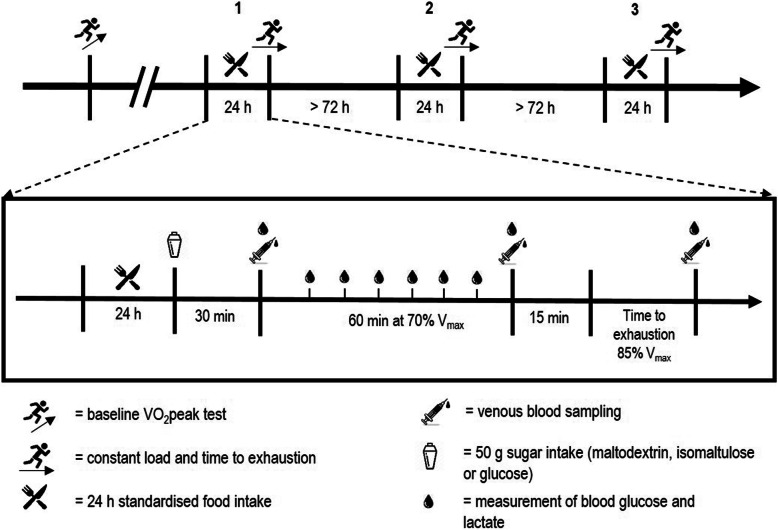
Experimental design

This double-blind randomized-crossover study consisted of four separate testing sessions: A preliminary ramp test to assess peak oxygen uptake (VO_2_peak) and maximal treadmill running speed (V_max_), as well as three separate experimental trials. The experimental trials were performed in a randomised and counterbalanced order and consisted of a 70-min constant workload test followed by a time to exhaustion test (TTE). The trials were performed after ingesting either isomaltulose, maltodextrin or glucose (Fig. 1). Randomisation was performed by technical staff not involved in data collection and both participants and test personnel were blinded to the experimental conditions. Blinding was only removed after data collection and analysis was completed.

### Performance testing

To assess V_max_ and VO_2_peak, a ramp test was performed on a treadmill with a starting inclination of 1%, to reflect the energetic cost of outdoor running [27]. After a 2 min warmup at 2.4 m·s^− 1^, the test started at 2.4 m·s^− 1^ and increased by 0.2 m·s^− 1^ every minute. If participants reached 5.2 m·s^− 1^, the incline was increased by 1° for each increment. Spirometric data was recorded breath by breath and interpolated for values for each second (Metalyzer® 3B; Cortex Biophysik GmbH, Leipzig, Germany), while heart rate (Polar H7 Sensor; Polar Electro, Kempele, Finland) was recorded every second. The spirometer was calibrated weekly with a reference gas (5% CO_2_ and 15% O_2_) and before each test with ambient laboratory air and with a 3-l syringe according to the manufacturer’s specifications. Participants were verbally encouraged to reach voluntary exhaustion. V_max_ was defined as the highest increment completed, while for additional degrees of inclination 0.2 m·s^− 1^ was counted. VO_2_peak was defined as the highest 30-s moving average oxygen uptake.

### Experimental trials

Nutritional intake was standardised by meal replacement 24 h prior to each trial, according to the recommendations of the German Society for Nutrition to allow for comparison between conditions. Nutrition was calculated upon a daily requirement of 35 kcal∙kg^− 1^ (fat: 0.9 g∙kg^− 1^, carbohydrates: 5.2 g∙kg^− 1^, protein: 1.5 g∙kg^− 1^), participants received 60.5 ± 4.7 g fat, 362.7 ± 31.4 g carbohydrates and 106.5 ± 9.7 g protein. Participants were also not allowed to take any over the counter supplements during the study period. Water was allowed ad libitum the day before and after the constant load trial. Testing was carried out in the morning and laboratory visits were separated by at least 72 h. Participants reported to the lab after an overnight fast and were provided with 50 g of either maltodextrin (100% Maltodextrin Carbs, Myprotein, Cheshire, UK), isomaltulose (Risulose, Evonik Creavis GmbH, Marl, Germany) or glucose (100% Glucose Carbs, Myprotein, Cheshire, UK) in 400 ml of water. This quantity was chosen according to recommendations previously outlined, showing that 50 g of isomaltulose was well gastrointestinally tolerated and did not alter gastric emptying rate [19], while larger amounts may reduce performance due to signs of gastrointestinal discomfort [12]. Similarly, during initial pilot testing in our lab, we found 50 g of isomaltulose to be well tolerated, while larger amounts led to strong symptoms of gastrointestinal discomfort and consequently to failing to completing the exercise session.

After 30 min, a first venous blood sample was drawn (pre) and the constant load trial commenced. The trials consisted of 10 min warm-up at 60% V_max_ followed by 60 min at 70% V_max_. Every 10 min the treadmill was stopped for 1 min for capillary blood sampling. Additionally, rate of perceived exertion (RPE) and gastrointestinal discomfort were recorded on a 1-10 scale. After the constant load test, another venous blood sample was taken (post). Following 15 min of passive recovery, participants performed a time to exhaustion test (TTE) at 85% V_max_ and a final venous blood sample was taken (pTTE). Spirometric data was recorded breath by breath and interpolated for each second for both tests (Metalyzer 3B, Cortex Biophysik GmbH, Leipzig, Germany). Fat and carbohydrate (CHO) oxidation rates were calculated for the constant load trial for each 10-min block from VO_2_ and VCO_2_ data according to the calculations by Peronnet and Massicotte (1991) [28].

### Blood sampling and analysis

Capillary blood samples (20 μl) were drawn from the earlobe into hemolyzing solution cups (EKF Diagnostic Sales, Magdeburg, Germany). Blood lactate and glucose concentrations were measured using the EKF Biosen S-Line Analyser (EKF Diagnostics GmbH, Barleben, Germany). Additionally, venous blood samples were drawn from the antecubital vein into serum separation tubes (BD, Plymouth, UK). After clotting for 10 min at room temperature, serum separation tubes were centrifuged at 1000 g at room temperature (Heraeus® Multifuge® 3 L-R, Kendro Laboratory Products, Newton, USA). Immediately after centrifugation, serum was separated into aliquots and stored at − 80 °C for further analysis. Serum insulin and glucose-dependent insulinotropic polypeptide (GIP) were assessed using the *Insulin ELISA Kit* (EIA-2935; DRG Instruments GmbH, Marburg, Germany) and *Human GIP (Total) ELISA Kit* (EZHGIP-54 K; Merck KGaA, Darmstadt, Germany). Samples were analysed in duplicate using a microplate reader (Multiscan™ FC; Thermo Scientific™, Waltham, USA) and the mean was used for statistical analysis.

### Calculations and statistical analysis

All data are presented as mean ± standard deviation (SD), apart from percent change, where mean and [95% confidence intervals] are reported. Statistical analysis was performed using SPSS 27.0 (SPSS, IBM Statistics, New York, US). Residual histograms, residual plots and Q-Q-plots were visually checked for homoscedasticity and normality prior to statistical analysis. Incremental area under the curve (AUC) was calculated for VO_2_, fat oxidation, CHO oxidation and RER using the trapezoid rule. Glucose fluctuation was calculated as the maximal difference in absolute glucose values assessed during the constant load trial (i.e. maximum glucose concentration - minimum glucose concentration). For better visualization, we additionally expressed the glucose fluctuation as percentage. Baseline differences (glucose, insulin, GIP) and differences between conditions (AUC VO_2_, AUC fat oxidation, AUC CHO oxidation, AUC RER, glucose fluctuation and gastrointestinal discomfort) were tested using a one-way analysis of variances (ANOVA). For time and interaction effects, a mixed factorial analysis of covariance (ANCOVA) was performed with Bonferroni correction for post-hoc tests. For this purpose, measurement times (i.e. minutes 0-70 during the constant load trial for blood glucose, VO_2_, fat and CHO oxidation rates and RER or pre, post and pTTE for insulin and GIP) were defined as within-group variables and isomaltulose, maltodextrin and glucose ingestion as between-condition variable. Effect sizes for main effects of the ANOVA and ANCOVA were reported as partial η^2^. To assess associations between changes in glucose, GIP and insulin concentrations across all conditions, Pearson product-moment correlation coefficients *r* were calculated. For all tests, statistical significance was accepted at *p* < 0.05.

## Results

### Spirometric data

**Fig. 2:**
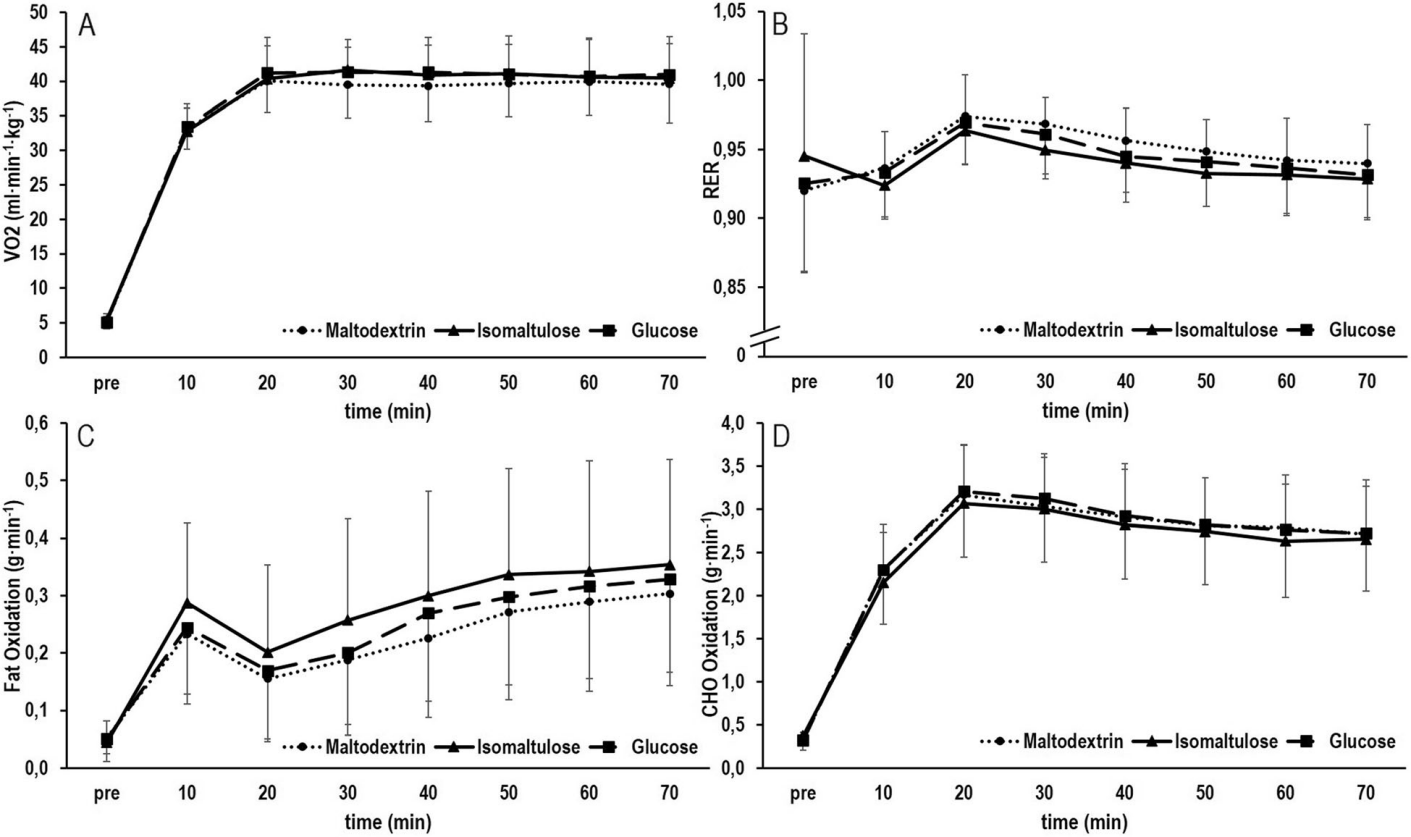
VO_2_ (**a**), respiratory exchange ratio (RER) (**b**), fat (**c**) and carbohydrate (CHO) oxidation (**d**) during the constant load trials with isomaltulose, maltodextrin and glucose, respectively

VO_2_ uptake, fat oxidation rate, carbohydrate (CHO) oxidation rate and respiratory exchange ratio (RER) are displayed in Fig. 2. Analysis of VO_2_, fat oxidation and CHO oxidation showed no main effect for time (VO2: *p* = 0.133, partial η^2^ = 0.034; fat: *p* = 0.087, partial η^2^ = 0.045; CHO: *p* = 0.745, partial η^2^ = 0.006) or interaction (VO2: *p* = 0.710, partial η^2^ = 0.027; fat: *p* = 0.208, partial η^2^ = 0.044; CHO: *p* = 0.685, partial η^2^ = 0.021). For RER, a main effect was observed for time (*p* < 0.001, partial η^2^ = 0.657) but not interaction (*p* = 0.248, partial η^2^ = 0.047). AUC of VO_2_ showed no difference between conditions (*p* = 0.806, partial η^2^ = 0.008). Similarly, the AUC of total fat oxidation (*p* = 0.576, partial η^2^ = 0.019) and CHO oxidation (*p* = 0.887, partial η^2^ = 0.004) did not differ between conditions. Furthermore, no between-condition difference was observed for AUC of RER (*p* = 0.529, partial η^2^ = 0.022).

### Blood glucose

**Fig. 3:**
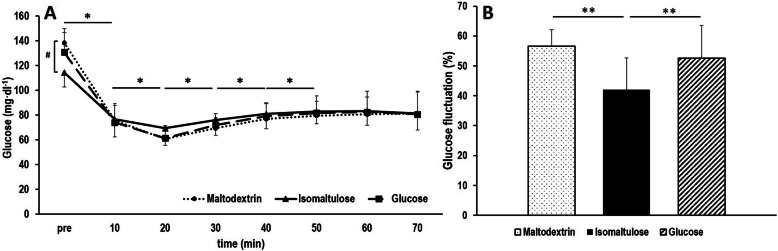
**a** Blood glucose concentrations during the constant load trial after ingestion of isomaltulose, maltodextrin or glucose. * statistical difference between sampling timepoints *p* < 0.05, # statistical baselines differences between conditions, *p* < 0.05. **b** Maximal glucose fluctuation (% difference between maximum and minimum glucose concentration) during the constant load trial after isomaltulose, maltodextrin and glucose ingestion, ** *p* < 0.001

Baseline postprandial blood glucose concentrations were lower by − 16.7% [− 21.8, − 11.6] following isomaltulose ingestion compared to maltodextrin (*p* < 0.001) and by − 11.5% [− 17.3, − 5.7] compared to glucose (*p* = 0.001) (Fig. 3a). Analysis of blood glucose concentrations showed a main effect for time (*p* < 0.001, partial η^2^ = 0.742) but not interaction (*p* = 0.872, partial η^2^ = 0.113). Baseline postprandial concentrations of blood glucose were higher compared to all other sampling points during the constant load trial (*p* < 0.001) in all conditions. Concentrations decreased from 0 to 10 min (*p* < 0.001), from 10 to 20 min (*p* < 0.001) and then increased from 20 to 30 min (*p* < 0.001), 30 – 40 min (*p* < 0.001) and 40 – 50 min (*p* = 0.029). After 50 min, blood glucose concentrations remained unaltered (*p* > 0.05) (Fig. 3a).

Furthermore, the maximal glucose fluctuation (Fig. 3b) differed between the conditions (maltodextrin: 56.7 ± 5.5 mg ∙ dl^− 1^, isomaltulose: 42.0 ± 10.7 mg ∙ dl^− 1^, glucose: 52.6 ± 10.9 mg ∙ dl^− 1^, *p* < 0.001, partial η^2^ = 0.395).

### Hormones

**Fig. 4:**
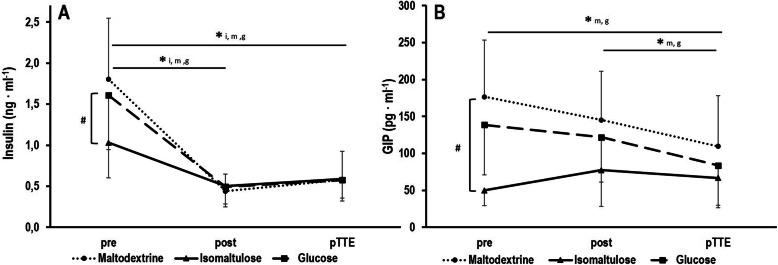
Serum concentrations of insulin (**a**) and glucose-dependent insulinotropic polypeptide (GIP) (**b**) after intake of isomaltulose, maltodextrin and glucose before (pre) and after a 70 min constant load trial (post) as well as after the time to exhaustion test (pTTE). * statistical differences between sampling timepoints for isomaltulose (i), maltodextrin (m) and glucose (g), # statistical baseline differences between conditions

Baseline postprandial insulin concentrations (Fig. 4a) were lower after isomaltulose ingestion by − 40.3% [− 50.6, − 30.0] compared to maltodextrin (*p* = 0.001) and by − 32.6% [− 43.9, − 21.2] compared to glucose (*p* = 0.012). Furthermore, a main effect was found for time (*p* < 0.001, partial η^2^ = 0.706) but not interaction (*p* = 0.503, partial η^2^ = 0.028). In all three conditions, insulin concentrations decreased over the constant load trial (all *p* < 0.001) and remained decreased throughout the TTE (all *p* < 0.001).

Baseline postprandial GIP concentrations (Fig. 4b) were reduced after isomaltulose ingestion by − 69.1% [− 74.3, − 63.8] compared to maltodextrin and by − 55.8% [− 70.7, − 40.9] compared to glucose (both *p* < 0.001). Furthermore, a main effect was observed for time (*p* < 0.001, partial η^2^ = 0.401) but not interaction (*p* = 0.244, partial η^2^ = 0.045). GIP concentrations decreased throughout the constant load test and TTE in both maltodextrin and glucose conditions (both *p* = 0.001). In isomaltulose, GIP remained unaltered throughout the exercise protocol (*p* > 0.05).

### Time trial performance

The mean time performed in the TTE was 9.22 ± 4.37 min, 8.70 ± 3.28 min and 9.25 ± 3.50 min for isomaltulose, maltodextrin and glucose, respectively. No between-conditions differences were observed (*p* = 0.876, partial η^2^ = 0.004).

### Gastrointestinal discomfort

**Fig. 5:**
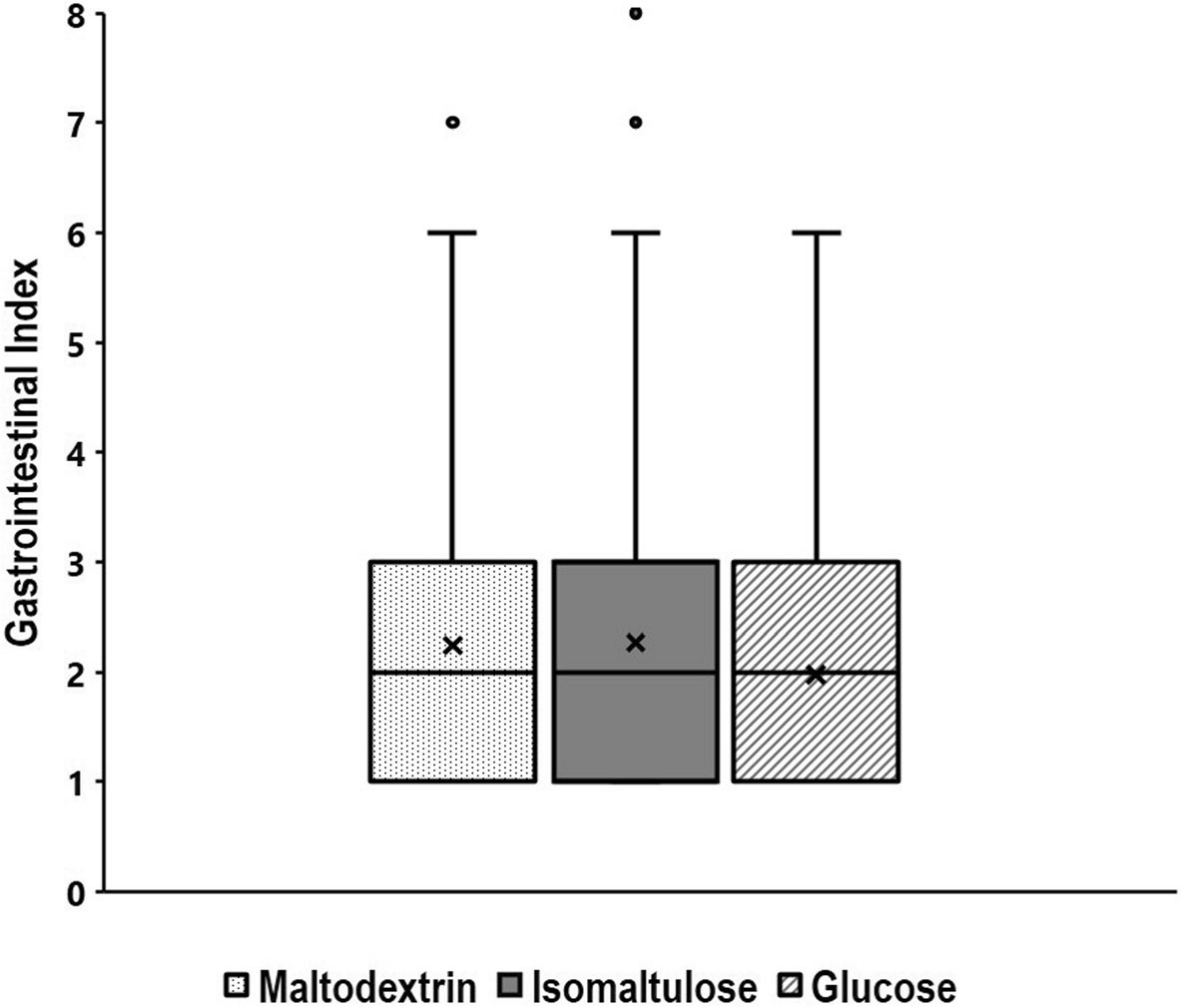
Subjective feelings of gastrointestinal discomfort during the constant load trials with isomaltulose, maltodextrin and glucose. Boxes extend from 25th to 75th percentile of each condition’s distribution, vertical extending lines denote adjacent values (i.e., the most extreme values within the interquartile range (IQR)), horizontal line in the box represents the median, x represents the mean value (maltodextrin: 2.24 ± 1.48, isomaltulose: 2.26 ± 1.65, glucose 1.98 ± 1.13), filled dots denote outliers (defined by 1.5 times IQR)

Subjective feelings of gastrointestinal discomfort (Fig. 5) did not differ between conditions (*p* = 0.119, partial η^2^ = 0.008).

## Discussion

The aim of this study was to assess the metabolic effects of isomaltulose ingestion prior to prolonged aerobic performance, when compared to ingestion of glucose and maltodextrin. We did not find differences between conditions in fat and CHO oxidation rates. However, isomaltulose ingestion led to lower baseline blood glucose, insulin and GIP concentrations and consequently lower fluctuations of these metabolites and hormones during and after exercise in comparison to glucose and maltodextrin. Lastly, running performance and subjective feelings of gastrointestinal discomfort did not differ between conditions.

In accordance with previous studies [13, 16, 19, 20], the ingestion of isomaltulose 30 min prior to a prolonged running protocol on the treadmill led to reduced postprandial blood glucose concentrations and reduced baseline insulin and GIP concentrations compared to glucose or maltodextrin ingestion. This has been suggested to be due to the slow cleavage of the α-1,6-glycosidic bond of isomaltulose, which almost completely bypasses the GIP releasing K-cells in the upper part of the intestine [10, 20]. However, the primary and novel aim of this study was to analyse the associations of glucose and hormone concentrations of insulin and GIP throughout prolonged endurance running. Interestingly, the initial between-condition differences in postprandial glucose concentrations were no longer observed during exercise. Similarly, no between-condition differences in insulin and GIP concentrations were existent. Considering the slower absorption of isomaltulose, similar blood glucose concentrations during exercise may be somewhat surprising. However, in healthy individuals approximately 90 - 95% of glucose appearing in circulation is rapidly taken up and metabolised [29] and possibly as a result no between-condition differences in blood glucose concentrations during exercise were observed.

During the constant load trial, insulin concentrations decreased in all conditions so that after 70 min of running between-condition differences were no longer observed. In contrast, while GIP concentrations seemed to be slightly reduced, they were not significantly decreased after the constant load trial. Therefore, it is likely that also other mechanisms may have affected insulin responses. For example, the glucagon-like peptide 1 (GLP-1) has previously been shown to be associated with insulin and glucose concentrations after ingestion of carbohydrates at rest [16, 20] but it remains to be assessed whether this is true for metabolic regulation during exercise as well. Nevertheless, after TTE in maltodextrin and glucose, insulin and GIP concentrations were both significantly decreased. Therefore, GIP is still likely to play somewhat of a regulatory role in regulating insulin and glucose concentrations during exercise after carbohydrate ingestion.

The reduced response of GIP along with lower glucose and insulin fluctuations after isomaltulose ingestion could be beneficial, as maintaining euglycemia is important for performance, for example for preserving glycogen stores and preventing fatigue [30, 31]. In fact in this study, hypoglycaemic blood glucose concentrations (defined as < 70 mg ∙ dl^− 1^ by the American Diabetes Association and European Medicines Agency) were only found in 20.4% of measurements after isomaltulose ingestion compared to 31.3 and 28.6% for maltodextrin and glucose ingestion, respectively. Further research should focus on the effects of isomaltulose ingestion on concentrations of glucose, insulin and GIP during exercise in clinical populations, such as diabetes or obesity, as there is often a higher hypoglaemic risk during exercise following a meal [32, 33].

When analysing the effects of different substrates on metabolic oxidation rates, no difference between conditions was observed for fat and CHO oxidation rates. This is in contrast to some previous studies. For example, König et al. (2016) observed increased rates of fat oxidation after ingesting 75 g of isomaltulose 45 min before cycling for 90 min at 70% VO_2_max [13]. Similar results were found by Oosthuyse et al. (2015) while cycling for 2 h at 60% of the peak power output and ingesting isomaltulose at 63 g ∙ h^− 1^ [12]. This discrepancy between our and previous findings may be explained by the lower dose of isomaltulose administered in our study (i.e. 50 g). This assumption is further supported by our data, as fat oxidation seemed to be somewhat higher after ingesting isomaltulose, even though this increase did not reach statistical significance (*p* = 0.087). Therefore, to increase fat oxidation rates during exercise, a larger amount of isomaltulose supplementation may be needed. Similarly, a greater amount of isomaltulose ingestion may also be necessary to acquire performance benefits. In the study by König et al. (2016) 75 g of isomaltulose ingestion resulted in an increased power output during a 16 km time trial performance [13], conversely our study along with others in cyclists and soccer players [14, 19, 22] found no differences in performance outcomes.

However, when ingesting isomaltulose during or before exercise, caution is necessary to avoid increased gastrointestinal discomfort, which is likely caused by the slower absorption rate and, therefore, an increased intestinal activity during exercise [10, 12]. Therefore, in this study 50 g of supplementation were chosen according to previous recommendations, showing this quantity not to affect gastrointestinal discomfort or gastric emptying rate compared to maltodextrin in cycling [19]. As running is more prone to causing symptoms of gastrointestinal distress due to greater bowel movements [26], the supplementation dose was adapted accordingly. However, we do acknowledge this as a possible limitation of this study. Supplementation was chosen to be safe and feasible so that participants could complete the trial, however larger amounts may be needed to obtain performance benefits. Thus, our findings need more practical validation and further research should aim to find the optimal dose of isomaltulose in direct comparative studies in order to provide firm recommendations for practitioners.

## Conclusion

In conclusion, 50 g isomaltulose ingestion seems to have a more advantageous effect on blood glucose, insulin and GIP response compared to maltodextrin and glucose, as was shown by reduced postprandial absolute concentrations and a lower rate of fluctuation during treadmill running exercise. However, glucose availability, as well as fat and carbohydrate oxidation rates remained unaffected. Furthermore, performance outcomes and gastrointestinal discomfort were not affected in this study but further research is required to offer specific guidelines on supplementing isomaltulose (amount, timing and frequency) in a performance setting.

## Data Availability

The datasets used and analysed during the current study are available from the corresponding author on reasonable request.

## Abbreviations

AUC: Area under the curve
CHO: Carbohydrates
GI: Glycaemic Index
GIP: Glucose-dependent insulinotropic polypeptide
RER: Respiratory exchange ratio
RPE: Rate of perceived exertion
T1DM: Type 1 Diabetes mellitus
T2DM: Type 2 Diabetes mellitus
TTE: Time to exhaustion test
V_max_: Maximal running speed
VO_2_max: Maximal oxygen consumption
VO_2_peak: Peak oxygen consumption
